# Eye care utilization among diabetics in the South African National Health and Nutrition Examination Survey (SANHANES-1): a cross-sectional study

**DOI:** 10.1186/s13104-020-05245-5

**Published:** 2020-08-31

**Authors:** Kwadwo Owusu Akuffo, Akosua Kesewah Asare, Ronel Sewpaul, Natisha Dukhi, David Ben Kumah, Eldad Agyei-Manu, Emmanuel Kofi Addo, Priscilla Reddy

**Affiliations:** 1 Department of Optometry and Visual Science, College of Science, Kwame Nkrumah University of Science and Technology, Kumasi, Ghana; 2 Health & Wellbeing, Human and Social Capabilities, Human Sciences Research Council, Cape Town, South Africa; 3 Usher Institute for Population Health Sciences and Informatics, College of Medicine and Veterinary Medicine, University of Edinburgh, Edinburgh, UK; 4 Department of Ophthalmology and Visual Sciences, Moran Eye Centre, University of Utah, Salt Lake City, Utah USA; 5 Department of Nutrition and Integrative Physiology, University of Utah, Salt Lake City, Utah USA; 6 Faculty of Health Sciences, Nelson Mandela University, Port Elizabeth, South Africa

**Keywords:** Eye care utilization, Diabetes, Population-based, South Africa, Sociodemographic characteristics, High-risk, Barriers, Disparities, Eye Care Services

## Abstract

**Objective:**

Diabetes is a chronic disease of uncontrolled blood sugar levels. People with diabetes are at an increased risk of developing visual impairment and other diabetes-related visual complications. The study aims to determine the eyecare utilization pattern and its associated determinants among diabetics in the South African National Health and Nutrition Examination Survey (SANHANES-1).

**Results:**

The mean age of participants was 56.2 years and 66.6% were females. The prevalence of eyecare utilization among participants was 49.0% and this differed significantly by age groups (p = 0.024) and the number of years since diabetes diagnosis (p < 0.001). After statistical adjustments, older age (55–64 years OR = 4.18, p = 0.003 and ≥ 65 years OR = 4.78, p = 0.002), having health insurance (OR = 6.32, p = 0.002), and having had diabetes for 6–10 years (OR = 4.23, p = 0.005) were significantly associated with eye care utilization. About half of people diagnosed with diabetes in South Africa have had an eye examination since diabetes diagnosis, which is disturbingly low given the impact of diabetes complications on eye health. Government policies must be directed at ensuring access to affordable health insurance and eye health education on diabetes.

## Introduction

Globally, diabetes and its complications are major public health problems [1], with an increasing burden of blindness and vision loss [2], as well as potential financial loss to individuals and nations [3]. People with diabetes have higher risks of developing cataracts, glaucoma, dry eyes and retinopathy [4–6] in comparison to the general population.

The South African National Health and Nutrition Examination Survey (SANHANES-1) was established as a nationally representative population health survey to address epidemiological transition and the changing health needs of South Africans [7]. The survey found that the prevalence of inpatient and outpatient health care utilization was 20.1% and 27% respectively, and further reported chronic conditions (including diabetes) as reasons for the utilization of the health care services [7]. Considering the adverse effects of diabetes on the eye, people with diabetes have a greater need for ophthalmic care and are expected to utilize eyecare services more frequently than the general population. These bring to fore the need to investigate the pattern of eye care utilization in this populace.

The purpose of this study therefore was: (1) to determine the eye care utilization patterns among people with diabetes in South Africa using data from SANHANES-1; and (2) to identify factors that influence the utilization of eye care services (i.e., sociodemographic or health-related factors correlates to eye care utilization). Secondary data analyses from this nationally representative study will contribute immensely to stakeholder policies on diabetic care and in general, eye care services and its delivery.

## Main text

### Participants and methods

#### Data and sample

This population-based (cross-sectional) study used data on self-reporting diabetic participants from the SANHANES-1 (a national survey conducted in 2011–2012). The survey used multi-stage disproportionate, stratified cluster sampling to select households within enumeration areas (EAs) stratified by province and locality types, where 10,000 occupied households were selected. Within the occupied households, 27,580 individuals of all ages were eligible to be interviewed and agreed to participate in the study. However, only 25,532 (92.6%) completed the interview. Of the latter number, 12,025 (43.6%) individuals volunteered to undergo a medical examination of which 7 455 were aged 15 years or older. A detailed description of the SANHANES-1 methodology has been previously reported [7].

Analyses were conducted on individuals aged ≥ 15 years who reported having been diagnosed with diabetes and who underwent the medical examination, where a clinician assessed their vision and measured their BMI and blood pressure. To investigate the association of all the predisposing, enabling and need factors on having had an eye examination, the analytic sample was based on individuals who had non-missing responses to all the independent variables.

#### Measures

Eye care utilization was assessed from participants’ responses to the following question: *“Since you were diagnosed with diabetes, have you ever had your eyes examined? This is an examination during which your pupils are usually dilated. It can make you temporarily sensitive to bright light.”* The individual factors associated with having an eye examination were investigated using Andersen’s Behavioural Model [8]. According to this model, variables were categorized into predisposing, enabling, and need factors.

#### Data analysis

Data were analysed in Stata 15.0. (StataCorp, Texas, USA 2016). The analyses applied sample weights to adjust for unequal probabilities of selection and nonresponse. The prevalence of having had an eye examination/s since diabetes diagnosis was presented by categories of the independent variables and difference between categories of a variable were tested using Chi square tests. Three multiple logistic regression models were used to investigate the predisposing, enabling and need factors associated with the outcome; having ever had an eye examination since being diagnosed with diabetes. Andersen’s model was applied in the multiple logistic regression models, where the predisposing, enabling and need factors were added sequentially. Model 1 included only the predisposing factors, Model 2, the predisposing and enabling factors and Model 3 included the predisposing, enabling and need factors.

## Results

### Description of the sample

Table 1 shows the demographic and lifestyle characteristics of the analytic sample (i.e. diabetics who underwent the physical examination including vision loss assessment with available data on all factors).

**Table 1:** Description of the analytic sample

	%	Frequency
Total	100.0	325
Age (mean, S.D.)	57.3	13.3
15–44	12.6	41
45–54	28.3	92
55–64	33.2	108
≥ 65	25.8	84
Sex		
Males	31.4	102
Females	68.6	223
Ethnicity		
African	62.5	203
White	2.2	7
’Coloured’(mixed race)	23.1	75
Indian	12.3	40
High risk alcohol use	11.7	38
Current smoker	14.5	47
Body mass index (BMI) (kg/m^2^) (mean, S.D.)	30.6	7.3
Underweight < 18.5 kg/m^2^	3.4	11
Normal weight 18.5–24.9 kg/m^2^	17.8	58
Overweight 25–29.9 kg/m^2^	26.5	86
Obese ≥ 30 kg/m^2^	52.3	170
Residence		
Rural	32.3	105
Urban	67.7	220
Has health insurance	17.5	57
Hypertensive	77.5	252
High cholesterol	23.4	76
Cardiovascular disease	20.0	65
Accessed health care in past 5 years	56.3	183
Clinician-assessed vision loss	29.8	97
Self-reported vision problems	43.4	141
Diabetic medication use		
No medication	16.3	53
Oral glycaemic medication	54.8	178
Insulin	3.7	12
Insulin and oral glycaemic medication	25.2	82
Number of years since diabetes diagnosis (mean, S.D.)	12.7	15.9
0–5 years	45.5	148
6–10 years	15.4	50
11–20 years	19.4	63
> 20 years	19.7	64

### Eye care utilization among people with diabetes

Overall, 49.0% of people with diabetes reported having ever had an eye examination since their diabetes diagnosis (Table 2). The prevalence of having an eye examination since diagnosis differed significantly by age groups (p = 0.024) and the number of years since diabetes diagnosis (p < 0.001). It was significantly higher among those with health insurance than those without (72.0% versus 43.8%, p = 0.003) and among those with hypertension than without (53.7% versus 36.6%, p = 0.034).

**Table 2:** Prevalence of having ever had an eye examination since diabetes diagnosis

	Had an eye examination since diabetes diagnosis			
	%	95% CI	n	p value
Overall	49.0	[41.4–56.6]	325	
Predisposing factors				
Age				
15–44 years	31.0	[16.3–50.8]	41	0.024
45–54 years	42.6	[30.4–55.7]	92	
55–64 years	59.6	[47.3–70.7]	108	
≥ 65 years	59.6	[45.3–72.4]	84	
Sex				
Males	50.0	[37.5–62.4]	102	0.857
Females	48.5	[39.3–57.8]	223	
Ethnicity				
African	49.1	[40.1–58.1]	203	0.678
’Coloured’(mixed race)	53.1	[38.8–67.0]	75	
White or Indian	41.6	[24.4–61.2]	47	
High risk alcohol use				
Low risk	49.0	[40.9–57.2]	287	0.996
High risk	48.9	[24.9–73.4]	38	
Current smoker				
No	50.8	[42.9–58.6]	278	0.257
Yes	37.5	[19.6–59.5]	47	
Body mass index (BMI)				
Underweight/normal weight: < 25 kg/m^2^	46.5	[31.5–62.1]	69	0.764
Overweight: 25–29.9 kg/m^2^	45.9	[32.7–59.7]	86	
Obese: ≥30 kg/m^2^	51.5	[41.1–61.8]	170	
Enabling factors				
Residence				
Rural	45.9	[34.4–57.9]	105	0.525
Urban	51.0	[41.2–60.7]	220	
Has health insurance				
No	43.8	[35.5–52.4]	268	0.003
Yes	72.0	[55.2–84.3]	57	
Need factors				
Hypertension				
No	36.6	[24.5–50.6]	73	0.034
Yes	53.7	[45.2–62.0]	252	
High cholesterol				
No	50.9	[42.3–59.4]	249	0.333
Yes	42.9	[29.8–57.1]	76	
Cardiovascular disease				
No	46.9	[38.8–55.2]	260	0.21
Yes	58.0	[42.0–72.4]	65	
Accessed health care in past 5 years				
No	47.2	[37.1–57.5]	142	0.658
Yes	50.4	[40.1–60.6]	183	
Clinician-assessed vision loss				
No	44.8	[36.0–53.8]	228	0.06
Yes	59.1	[46.4–70.7]	97	
Self-reported vision problems				
No	47.4	[37.2–57.8]	184	0.628
Yes	51.2	[40.2–62.1]	141	
Diabetic medication use				
No medication	35.2	[20.8–53.0]	53	0.056
Oral glycaemic medication	46.6	[36.2–57.3]	178	
Insulin	64.3	[20.7–92.6]	12	
Insulin and oral glycaemic medication	66.9	[54.1–77.6]	82	
Years since diabetes diagnosis				
0–5 years	33.0	[23.9–43.5]	148	< 0.001
6–10 years	68.2	[50.4–81.9]	50	
11–20 years	66.0	[49.3–79.5]	63	
> 20 years	64.4	[48.8–77.4]	64	

OR, odds ratio; CI, confidence interval; n, number of participants; ref, reference group

### Factors associated with eye care utilization among people with diabetes

Model 1, with predisposing factors (Akaike’s information Criteria (AIC): 750482.6, Prob > F = 0.112), showed that older age (55-64 years, odds ratio (OR) = 3.77, p = 0.007 and ≥ 65 years OR = 3.68 p = 0.011 compared with 15-44 years) was significantly associated with having had an eye examination (Table 3).

**Table 3:** Multiple logistic regression of having an eye examination since diabetes diagnosis and related factors

	Model 1			Model 2			Model 3		
	OR	95% CI (OR)	p-value	OR	95% CI (OR)	p-value	OR	95% CI (OR)	p-value
Predisposing factors									
Age									
15–44 years	ref	–	–	ref	–	–	ref	–	–
45–54 years	1.82	[0.66–5.00]	0.243	1.62	[0.6–4.35]	0.338	2.00	[0.77–5.21]	0.154
55–64 years	3.77	[1.45–9.77]	0.007 *	4.58	[1.89–11.06]	0.001 *	4.18	[1.63–10.72]	0.003 *
≥ 65 years	3.68	[1.35–10.02]	0.011 *	5.90	[2.2–15.82]	<0.001 *	4.78	[1.75–13.05]	0.002 *
Sex									
Males	ref	–	–	ref	–	–	ref	–	–
Females	0.81	[0.41–1.6]	0.546	0.89	[0.43–1.84]	0.753	0.95	[0.45–2.02]	0.897
Ethnicity									
African	ref	–	–	ref	–	–	ref	–	–
’Coloured’(mixed race)	1.71	[0.75–3.92]	0.204	1.91	[0.75–4.87]	0.173	1.91	[0.72–5.07]	0.192
White or Indian	0.65	[0.28–1.48]	0.299	0.29	[0.09–0.98]	0.046 *	0.30	[0.09–1.05]	0.060
High risk alcohol use	1.29	[0.35–4.76]	0.698	0.98	[0.27–3.51]	0.973	1.50	[0.43–5.29]	0.523
Current smoker	0.50	[0.19–1.31]	0.155	0.45	[0.17–1.24]	0.123	0.39	[0.15–1.04]	0.060
Body mass index (BMI)									
Underweight/normal weight: < 25 kg/m^2^	ref	–	–	ref	–	–	ref	–	–
Overweight: 25–29.9 kg/m^2^	0.74	[0.32–1.68]	0.467	0.40	[0.17–0.96]	0.041 *	0.38	[0.15–0.93]	0.034 *
Obese: ≥ 30 kg/m^2^	1.05	[0.47–2.35]	0.907	0.75	[0.33–1.72]	0.495	0.77	[0.32–1.85]	0.563
Enabling factors									
Residence									
Rural				ref	–	–	ref	–	–
Urban				1.44	[0.74–2.82]	0.283	1.41	[0.68–2.91]	0.355
Has health insurance				6.98	[2.69–18.15]	<0.001 *	6.32	[2.00–19.91]	0.002 *
Need factors									
Hypertensive							1.06	[0.54–2.08]	0.868
High cholesterol							0.64	[0.3–1.38]	0.253
Cardiovascular disease							1.33	[0.6–2.94]	0.479
Accessed health care in past 5 years							0.98	[0.52–1.85]	0.949
Clinician assessed vision loss							1.97	[0.83–4.69]	0.125
Self–reported vision problems							0.52	[0.24–1.15]	0.106
Diabetic medication use									
No medication							ref	–	–
Oral glycaemic medication							0.99	[0.39–2.48]	0.977
Insulin							2.67	[0.47–15.2]	0.267
Insulin and oral glycaemic medication							2.57	[0.9–7.35]	0.077
Number of years since diabetes diagnosis									
0–5 years							ref	–	–
6–10 years							4.23	[1.55–11.54]	0.005 *
11–20 years							2.72	[0.99–7.43]	0.052
> 20 years							2.38	[0.91–6.2]	0.076

OR, Odds ratio; CI, confidence interval; n, number of participants; ref, reference group; *, statistically significant p values (p < 0.05)

In Model 2 (AIC: 692682.6, Prob > F = 0.001), which included predisposing and enabling factors, the associations with older age were similar to that of Model 1. White or Indian ethnicity (OR = 0.29, p = 0.046 compared with African ethnicity) and being overweight (OR = 0.40, p = 0.041 compared with normal-weight or underweight) were associated with significantly reduced odds of having had an eye examination. Having health insurance (OR = 6.98, p < 0.001) was significantly associated with increased odds of having had an eye examination.

The final adjusted Model 3 (AIC: 654052.6, Prob > F = 0.008), included predisposing, enabling and need factors. Older age (55–64 years OR = 4.18, p = 0.003 and ≥ 65 years OR = 4.78, p = 0.002 compared with 15–44 years), having health insurance (OR = 6.32, p = 0.002), and having had diabetes for 6–10 years (OR = 4.23, p = 0.005 compared with 0-5 years) were significantly associated with having had an eye examination. However, being overweight (OR = 0.38, p = 0.034 compared with normal-weight or underweight) was significantly associated with reduced odds of having had an eye examination.

## Discussion

This study presents data on the pattern of utilization of eye care services among people with diabetes in South Africa using data from a population-based national survey (SANHANES-1). Almost half of the persons who self-reported diabetes had accessed eye care services since diabetes diagnoses. Older age, having health insurance and duration of diabetes were associated with an increased likelihood of having an eye examination in this study. However, being overweight was associated with a decreased likelihood of having an eye exam among participants with self-reported diabetes.

The rates of eye care utilization among people with diabetes have been reported in different population-based studies [9–12]. For example, in the United States, both Benoit et al. [9] and MacLennan et al. [13] reported that nearly half of all patients with diabetes had not had an eye exam in over 5 years. In a hospital-based study in India, Sreenivas et al. [12] reported that about a third of diabetic patients sampled had accessed eye care services in the last 1 year. In Tanzania, Mumba et al. [11] reported that about 60% of diabetic patients sampled had undergone a dilated eye exam after their diagnosis. It is clear that the pattern of utilization varies in different countries and the barriers or factors that promote utilization are also varied.

Consistent with previous studies [9, 13, 14], older age was associated with eye care utilization among people with diabetes. Specifically, people aged 55 years and above were more likely to utilize eye care services. This may be attributed to the compounding effects of increasing resistance to insulin and dysfunction of the pancreatic islets with aging [15], thus diabetes often affects older people more than young people [16].

Having health insurance influences eye care utilization among people with diabetes [13, 17]. For instance, Mier et al. [18] reported that people with diabetes with medical insurance cover were five times more likely to utilize eye care services than those who did not have insurance. Contrary to most studies (including ours), a study in China reported that the absence of medical insurance cover does not serve as a barrier for eye care utilization among diabetes [10]. Diabetic eye exams in China are affordable and so the availability or otherwise of medical insurance is not a predictive factor for uptake of eye care services [10]. South Africa has both private and public health insurance systems. The public health insurance system primarily serves a large proportion of the population, but like in most developing countries, it is continually underfunded and understaffed. The Medical Aid Schemes Act introduced in 2004 [19] recommends that all medical aids are required by law to cover the cost of treatment and care of diabetes. These benefits, known as Prescribed Minimum Benefits, ensure that members of schemes have access to minimum health services.

Diabetes duration is also a major factor in eye care utilization among people with diabetes. We found that diabetes duration of 6–10 years was significantly associated with increased uptake of eye examination after adjusting for confounders. Longer diabetes duration is known to be associated with increased prevalence/incidence of retinopathy [20, 21] and thus an increased need for an eye examination by an eye care professional. It is recommended that persons with diabetes perform a yearly eye examination. The Ophthalmological Society of South Africa’s (OSSA) recommended guideline for a diabetic eye exam is that every patient must have a dilated retinal examination at least once every year to screen for diabetic retinopathy [22].

We found that being overweight was a barrier to eye care service utilization. This unanticipated finding, however, has been previously reported by Baumeister et al. [17] who found poor eye care service utilization among overweight people due to very minimal physical activity. Sedentary lifestyle, particularly in individuals whose work requires many hours seated and working on computers, has been associated with obesity, which in turn may lead to diabetes. Lack of exercise and physical activity might even form barriers to consulting with a physician. Overweight persons may be discouraged to seek care because of numerous factors. Among these are inadequate medical equipment (e.g.: small/ill-fitting blood pressure cuffs), and dismissive attitudes among healthcare professionals in the form of attribution of all medical problems to weight.

In conclusion, should our sample be representative of the population; our findings suggest that less than half of the diabetic population in South Africa are accessing eye care services. This is worryingly low given the impact of diabetes on vision.

## Limitations

Our study had a few limitations. Firstly, the data on eye exam since diagnosis, self-reported cardiovascular disease conditions, and cholesterol may be subject to recall bias. Also, hypertension was assessed by self-report as well as blood pressure measures obtained in the physical exam. A second limitation of our study design was that, the percentage of patients who had been utilizing eye care is likely to be underestimated. Based on the international guidelines for diabetic eye exam which requires that people with type 1 diabetes must have annual examinations, beginning 5 years after the onset of their disease while those with Type 2 diabetes should have a prompt examination at the time of diagnosis. However, we only assessed eye care utilization based on whether a participant had ever had an eye examination in the last 1 year without taking into cognizance the number of years since diabetes was diagnosed.

## Data Availability

The dataset(s) supporting the conclusions of this article is (are) available on request from the Human Sciences Research Council (HSRC) data research repository via access dataset http://www.hsrc.ac.za/en/research-data/.

## Abbreviations

HSRC: Human Sciences Research Council
REC: Research Ethics Committee
SANHANES: South African National Health and Nutrition Examination Survey
WHO: World Health Organization
